# Potential Impact of Influenza A/H1N1 Pandemic and Hand-Gels on Acute Diarrhea Epidemic in France

**DOI:** 10.1371/journal.pone.0075226

**Published:** 2013-10-04

**Authors:** Pascal Crépey, Mathilde Pivette, Moïse Desvarieux

**Affiliations:** 1 École des hautes études en santé publique Rennes, Sorbonne Paris Cité, Paris, France; 2 UMR EPV Emergence des Pathologies Virales –190, Aix-Marseille University, Marseille, France; 3 MAP5, Université René Descartes-Paris5, Paris, France; 4 Department of Epidemiology, Mailman School of Public Health, Columbia University, New York City, New York, United States of America; 5 U 738, Institut National de la Santé et de la Recherche Médicale, Paris, France; University of Hong Kong, Hong Kong

## Abstract

**Background:**

The 2009 A/H1N1 influenza pandemic has received a great deal of attention from public health authorities. Our study examines whether this pandemic and the resulting public health measures could have impacted acute diarrhea, a prevalent, highly transmissible and historically monitored disease.

**Methods:**

Using augmentation procedures of national data for the previous five years (2004–2009), we estimated the expected timing and incidence of acute diarrhea in France in 2009–2010 and evaluated differences with the observed. We also reviewed national hand gels for the same period.

**Findings:**

Number of episodes of acute diarrhea in France in 2009–2010 was significantly lower than expected until the third week of December (−24%, 95% CI [−36%; −9%]), then significantly higher (+40%, 95% CI [22%; 62%]), leading to a surplus of 574,440 episodes. The epidemic was delayed by 5 weeks with a peak 1.3 times higher than expected. Hand-gels sales inversely correlated with incidence of both influenza-like illness and acute diarrheal disease. Among individuals >65 yo, no excess cases of influenza and no excess rebound in acute diarrhea were observed, despite similar delay in the onset of the seasonal diarrheal epidemic.

**Interpretation:**

Our results suggest that at least one endemic disease had an unexpected behavior in 2009–2010. Acute diarrhea seems to have been controlled during the beginning of the pandemic in all age groups, but later peaked higher than expected in the younger population. The all-age delay in seasonal onset seems partly attributable to hand-gels use, while the differential magnitude of the seasonal epidemic between young and old, concurrent for both influenza and acute diarrhea, is compatible with disease interaction.

## Introduction

The first cases related to a novel strain of Influenza A/H1N1 appeared in Mexico within the first months of 2009, resulting in the first influenza pandemic of the 21^st^ century [1]. During the international crisis that followed, a number of containment measures were taken at the national [2], [3] and international levels [4]. While some measures were specific to influenza (e.g. mass vaccinations campaigns, recommended by WHO and initiated in October 2009 in many countries including France [5]), a number of non-pharmaceutical measures able to impact communicable disease transmission generally were also variably recommended across communities: restrictions on mass transport services and nonessential travels [4], social distancing interventions (closing schools and workplaces and limiting public gatherings) [4], [6], and avoiding contact with the sick. At the national level, the most universally recommended two-set measures were frequent hand-washing and availability and use of alcohol-based hand sanitizers; as well as covering sneezes/coughs, and the use of masks, all widely advocated through extensive print, audio, video campaigns conducted throughout virtually all public spaces, airwaves and outlets. Recent theoretical models confirm the legitimacy of these early, sustained, and non-pharmaceutical interventions for influenza containment[7]–[12]. However, there is a severely lacking amount of information about the potential indirect impact of this international crisis on other prevalent diseases. The aim of this study was to take advantage of the unique natural experiment of large-scale multi-focal public health interventions and to assess their impact on a historically monitored commonly transmitted infectious disease.

## Methods

We hypothesized that the collateral effect of the previously described large-scale interventions would reduce transmission of acute diarrheal disease in France.

Acute gastro-enteritis was chosen because it is among the most common illnesses worldwide, with more than 4.5 M cases in France in 2011 (data from French *Sentinelles* Network). It is also highly transmissible with two main etiologic agents, rotavirus for children<2 years and Norovirus for adults [13], requiring small infectious doses [14].

Our analysis is based on syndromic data from the *Sentinelles* network database [15]–[17]. *Sentinelles* is a network of 1300 volunteer general practitioners working in metropolitan regions throughout France (see http://www.sentiweb.org/). They continuously report cases of eight different diseases. In particular, the database includes weekly reports of influenza like illness since 1984 and of acute diarrhea cases since 1990. A case of syndromic acute diarrhea was defined as a patient having at least three daily, soft or watery stools in the past 14 days. In order to analyze influenza incidence in France, we used the estimated number of Influenza-like illness cases (ILI, defined as patients with sudden fever >39°C with myalgia and respiratory signs) reported by the *same* network. This database has recently been used to show the impact of school closures –another kind of preventive measure– on influenza epidemic [17]. We acknowledge that ILI is a poor estimator of true influenza incidence. However, we are more interested in this study by the timing of the epidemic and by yearly comparisons than by the true level of influenza incidence.

We then estimated the expected weekly incidence, or number of cases, of acute diarrhea, and influenza during the 2009–2010 period. Data augmentation procedures (bootstrapping) were used to generate 1000 new datasets of weekly incidence, or number of cases, based on the five previous years (2004–2009). A 5% random noise was added to the data to account for potential inaccuracy in data collection. Thanks to this method we were able to compute a weekly average incidence and 95% bootstrap confidence intervals. Data interpolation was performed using a sliding average to remove noise in the data and highlight the general trend. This simple approach can be qualified as a non-adaptive version of the *“analogues method”* previously used on similar data [18], i.e. based on the basic premise that an epidemic is likely to behave as previous ones. To validate the method we estimated the 2008–2009 “normal” season of acute diarrhea epidemic using preceding years.

Observed and expected 2009/2010 values of incidence rates, number of cases, time taken to reach epidemic peak, and peak height were compared. The epidemic period was determined using the Serfling method [19].The Serfling method uses cyclic regression to model seasonal pattern of incidence and to define epidemic threshold. We also conducted separate analysis for the >65years-old.

We chose to analyze alcohol-based hand gels because of their sustained recommendation at the national level, and their potential to interfere with fecal-oral transmission via chemical cleansing of the hands. Analysis of the evolution of hand gels sales in France is based on data from a stratified sample of 3004 pharmacies on the French metropolitan territory set up by the company Celtipharm [20]. These pharmacies automatically report their sales continuously several times a day since 2007. Thanks to a regularly updated exhaustive database of the 22,458 active French pharmacies, a stratification to improve representativeness is performed on sales revenue (6 levels for global revenue and 4 levels per type of sales: prescribed drugs, OTC, and other type of sales), localization (5 geographic areas) and sales area (5 types, from rural to densely urban). Each stratum has a minimum of 30 pharmacies or is merged with neighboring strata. Sampling rates per strata are computed with the Neyman optimal allocation algorithm [21]. Extrapolations from the sample have been validated with data from drug manufacturers who distribute their products directly (and only) to pharmacies.

## Results

**Figure 1:**
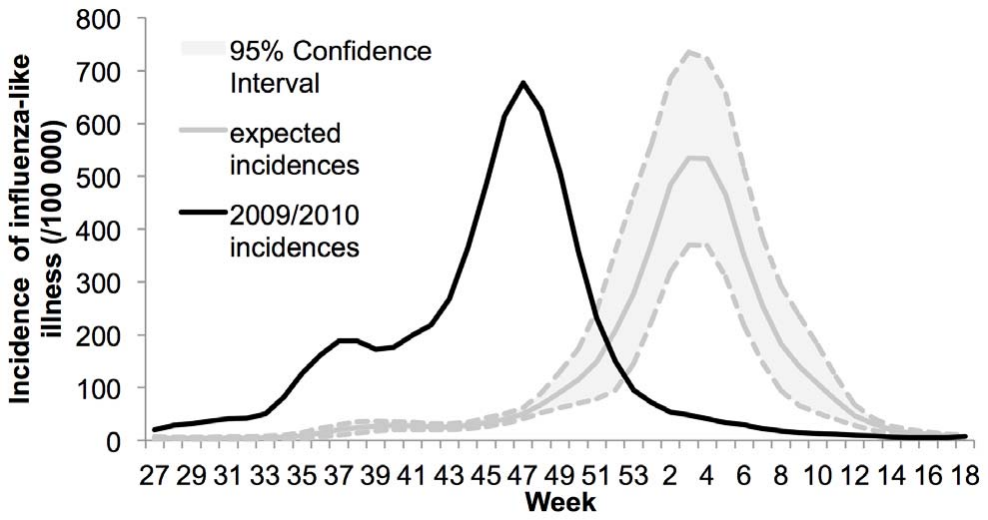
Incidences of influenza-like illness in 2009–2010 for France. Observed incidences are shown with black lines while expected incidences are shown with gray lines along their 95% confidence interval in dashed lines. Expected incidence is based on 2004–2009 data. The comparison shows the pandemic arrived sooner than previous years. The plot show smoothed data.

The 2009 influenza pandemic in France was different from previous seasonal influenza epidemics, both in timing and magnitude (Figure 1). The epidemic started in July 2009, with a peak in the middle of November, and ended in January 2010. In previous years (2004 to 2009), the epidemic lasted from November to April, with a peak in January (Figure S1). The 2009 epidemic peak was also higher than during previous seasonal influenza epidemics.

**Figure 2:**
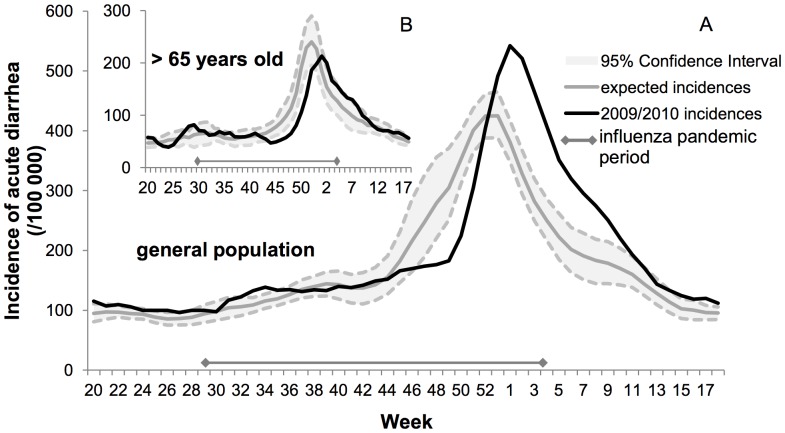
Incidences of acute diarrhea in France in 2009–2010. **a)** Observed incidence of acute diarrhea in general population (black line) peaks later and higher than the corresponding expected incidence (gray line, 95% confidence interval shown in dashed lines). **b)** In patients >65 years old, the peak is also delayed but its maximum is lower than expected. The interval shown at the bottom of the plots stands for the pandemic period in France defined by the Serfling method. Both plots show smoothed data.

**Figure 3:**
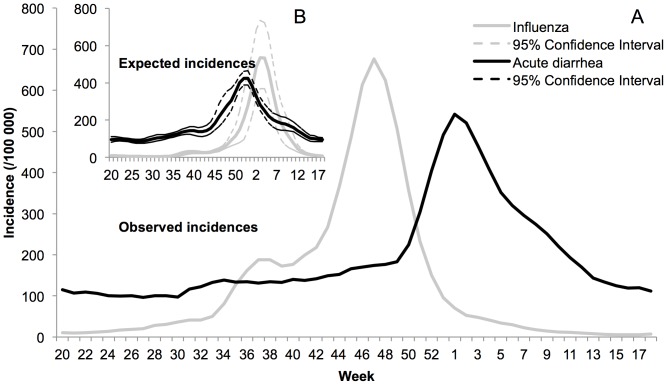
Comparison between influenza (gray line) and acute diarrhea (black line) incidence patterns in France. **a)** Observed incidences in 2009–2010 show that influenza epidemic preceded the acute diarrhea epidemic. During the preceding years the acute diarrhea epidemic was arriving first, as shown on **b)** with the expected incidences based on 2004–2009 data. Both plots show smoothed data.

In 2009–2010, the number of acute diarrhea episodes in France was significantly lower than expected until the third week of December (−24%; 95% CI: −36%, −9%), and then significantly higher (+40%; 95% CI: 22%, 62%), leading to an excess of 574,440 episodes (+14%; 95% CI: −2%,+34%) (Table 1). As shown in Figure 2, the epidemic started the second week of December and was delayed by five weeks; the maximum incidence occurred during the first week of January, one week later than expected, with the peak 1.3 times higher. Details of the differences between estimated and observed incidence are in Table 1. Figure 3 and S2 show that the beginning of the acute diarrhea epidemic in previous years (2004–2009) was observed 3 weeks *before* the beginning of the influenza epidemic, with an epidemic peak one month before the peak of influenza. In 2009–2010, however, the acute diarrhea epidemic started right *at the end* of the influenza epidemic, with a peak six weeks after the peak of influenza.

**Table 1:** Comparison between 2009–2010 observed acute diarrhea incidence and the expected incidence estimated from previous years (2004–2009) in France.

Differences with 2009–2010 incidence		low incidence	expected incidence	high incidence
**Period between weeks 44 and 52**	*Incidence (/100 000)*	−209	−**608**	−1,104
	*Number of cases*	−134,124	−**391,297**	−710,282
	*% of increase or decrease*	−9%	−**24%**	−36%
**Period between weeks 53 and 18**	*Incidence (/100 000)*	+2,019	**+1, 501**	+935
	*Number of cases*	+1,298,442	**+965,737**	+601,107
	*% of increase or decrease*	+62%	**+40%**	+22%
**Entire studied period between** **weeks 44 and 18**	*Incidence (/100 000)*	+1,810	**+893**	−170
	*Number of cases*	+1,164,318	**+574,440**	−109,175
	*% of increase or decrease*	+34%	**+14.1%**	−2%

Low and high incidence scenarios are given by the bound of bootstrapped 95% confidence interval. The studied period is divided in two sub-periods, before and after the week 52 when the observed and expected incidences intersect.

Estimations of the 2008–2009 “normal season” using the same method based on 2004–2008 years or using a larger dataset (2000–2008) were consistent with observations (start and peak of the epidemic in the 95% confidence interval, see figures S3 and S4 for details).

Because the 2009–2010 Influenza A/H1N1 primarily affected the young, sparing the old, we decided to separate the >65 years-old to determine whether both the delay in onset and the higher magnitude rebound were observed in this age group. While the onset of the acute diarrhea seasonal epidemic was delayed, no excessive rebound was observed among older subjects (Figure 2b): the number of episodes in 2009/2010 was significantly lower than expected until the last week of December (−19%; 95% CI: −25%, −8%), a deficit which was never recouped, leading to an overall reduction of 11% in the total number of cases.

**Figure 4:**
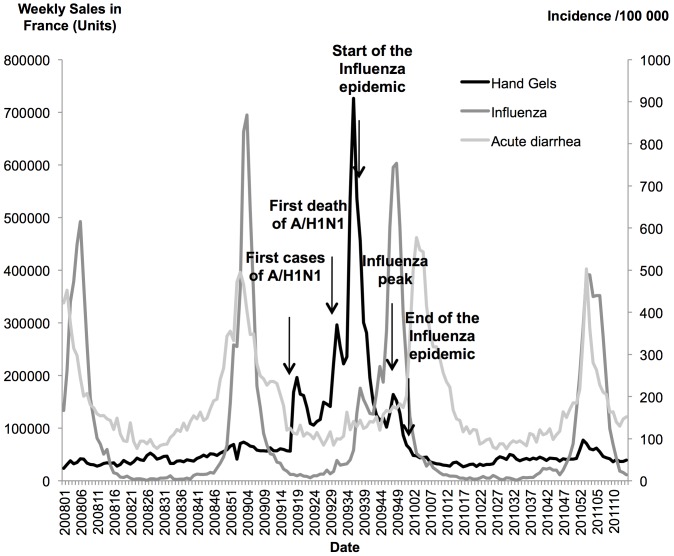
Weekly incidences of acute diarrhea and influenza-like-illness, along with weekly sales of hand gels in France from January 2008 to March 2011. We observe inverse correlations between sales of hand-gels, and incidence of influenza-like-illness and acute diarrhea. We also observe no hand-gels sales increase during the two epidemic seasons (2007–2008, 2008–2009) preceding the pandemic and the one following it (2010–2011).

Sales of hand gels peaked just before and during the very early beginning of the influenza epidemic but plummeted right before the real beginning of the epidemic peak (Figure 4). In addition, the acute diarrhea epidemic started when the sales were at their lowest level. Of note, we also observe no hand-gels sales increase during the two epidemic seasons (2007–2008, 2008–2009) preceding the pandemic and the one following it (2010–2011) (Figure 4).

## Discussion

We report unusual incidence patterns of acute diarrhea associated with the 2009–2010 influenza A/H1N1 pandemic and accompanying public health measures in France. Although we postulated a positive impact of non-pharmaceutical measures (notably alcohol-based hand-gels) on this communicable disease, the dramatic rebound in number of cases after the pandemic crisis was surprising. Our estimates translate into a 24% decrease in the number of acute diarrhea during the influenza pandemic followed by a 40% increase afterwards, which corresponded to more than 570,000 additional diarrheal cases compared to previous years.

Thus, overall, we observed two phases: a disease control period during the influenza pandemic peak (translating into a delayed onset), then an epidemic rebound afterwards (translating into a higher peak magnitude). Regarding the first phase, at least two hypotheses can be offered. First, non-pharmaceutical interventions such as hand-gels may have had an impact on other communicable diseases transmitted by inter-individual contacts, thereby controlling acute diarrhea. Second, and perhaps concomitantly, competition between infections could have occurred, in which people already affected by influenza were more likely to reduce their contacts, hence affecting the pool of susceptibles for other communicable diseases. Consequently, the rebounding second stage may have coincided with a letdown in the preventive measures that were used in the first stage. An increased seasonal effect could also explain the higher magnitude of the later acute diarrhea epidemic: the epidemic arriving later in the winter season may have benefited from a higher impact of the weather on the force of infection (lower temperature, higher precipitation, etc…).

Alternatively, however, the population may have also been weakened by the influenza pandemic since this particular strain has been shown to trigger impaired immune response which may result in higher susceptibility [22]. However data from our surveillance network show that the influenza incidence rate in subjects 65 years and older was no different than in previous years [23]. In addition it seems that aged individuals developed a milder form of the disease compared to previous years and contrary to the younger population [24]. This population is critical in differentiating the impact of universally recommended public health measures from inter-disease interaction. Indeed, subjects older than 65 years old in France only experienced the delayed onset (potentially consistent with increased hand-gels use), but not the higher-than-expected rebounding stage in acute diarrheal disease. This discrepancy between delay with no higher magnitude, specific to that age group less affected than the younger population, could reveal a link between these two diseases in the young population, beyond a let down of public health measures. Not being as affected as the younger, the older population had no acute diarrhea rebound. The interrelated nature of diseases has been proposed several times [25]–[27] to explain for example the relationship between the 1918 influenza pandemic and declining death rates of tuberculosis, cardiovascular disease and nephritis in the US [28], [29]. Here, excess cases of acute diarrhea could be explained as a “temporary harvesting effect” triggered by the influenza pandemic. All the above could fit diseases interaction models of syndemics [30] or pathocenosis [31], defined as the idea of complete interaction between diseases in an interrelated ecosystem.

Reporting bias could also have affected our data. An underreporting of acute diarrhea during the influenza pandemic could explain the sharp increase in the number of cases after the crisis. Furthermore, differential diagnosis could have been a major issue considering 38% of patients with influenza A/H1N1 presented with symptoms of nausea and diarrhea [32]. An alternative explanation could be the priorities, explicit or implicit, in which diseases were given treatment during the pandemic. In contrast, an over-reporting of acute diarrhea after the pandemic could partly explain the higher observed peak. For example, an increase in care seeking behavior may have occurred after the pandemic. However, these biases could explain some amplitude variations but not the observed epidemic delay of 6 weeks nor is there any easily identifiable reason why they would have spared the >65 years-old, all the more vulnerable to diarrhea-induced dehydration.

Hand gel sales analysis may present different bias. First, we don’t know whether people who bought hand gels effectively used it. Health seeking behaviors vary by demographic, social and economic factors. Population consulting a general practitioner and buying health products may differ. Moreover, it is difficult to link a number of hand gel sales to a number of patients. However, the sales analysis is useful to determine temporal evolution and to detect abnormal phenomena.

## Conclusion

Our results tend to show that the 2009–2010 acute diarrhea epidemic has been delayed compared with previous years. This delay is temporally associated with a peak of hand-gel sales, which could be a proxy for other control measures, and with the influenza pandemic peak. However, the nature of their associations is still unclear since it could be causal, or simply fortuitous. But the temporal proximity, the social or possibly biological arguments emphasize the need for further studies to clarify the nature of the link. Its existence would highlight the complexity of predicting the behavior of an epidemic without consideration to the ecological niche since delaying an epidemic may not imply decreasing the number of cases.

## Supporting Information

Figure S1 **Incidences of influenza-like illness in 2009–2010 and in the previous year (2004–2009) for France.** Observed incidences of influenza in 2009–2010 (black lines) arrived sooner than previous years. The plot shows smoothed data.
(PDF)

Figure S2 **Incidences of acute diarrhea in 2009–2010 and in the previous year (2004–2009) in France.** Observed incidence of acute diarrhea in 2009–2010 (black line) peaks later and higher than the incidence from previous years. The plot shows smoothed data.
(PDF)

Figure S3 **Incidences of acute diarrhea in 2008–2009 for France.** Observed incidences are shown with black lines while expected incidences are shown with gray lines along their 95% confidence interval in dashed lines. Expected incidence are based on 2000–2008 data. The comparison shows that estimations of the 2008–2009 incidences are consistent with observations. The plot shows smoothed data.
(PDF)

Figure S4 **Incidences of acute diarrhea in 2008–2009 for France.** Observed incidences are shown with black lines while expected incidences are shown with gray lines along their 95% confidence interval in dashed lines. Expected incidences are based on 2004–2008 data. The comparison shows that estimations of the 2008–2009 incidences are consistent with observations. The plot shows smoothed data.
(PDF)
