# Supplementary material for: Potential Impact of Influenza A/H1N1 Pandemic and Hand-Gels on Acute Diarrhea Epidemic in France
Source: PLoS One. 2013 Oct 4;8(10):e75226. doi: 10.1371/journal.pone.0075226 (PMC3790785; doi:10.1371/journal.pone.0075226)
Supplement: Figure S1 — Incidences of influenza-like illness in 2009–2010 and in the previous year (2004–2009) for France. Observed incidences of influenza in 2009–2010 (black lines) arrived sooner than previous years. The plot shows smoothed data. (PDF) [file pone.0075226.s001.pdf]

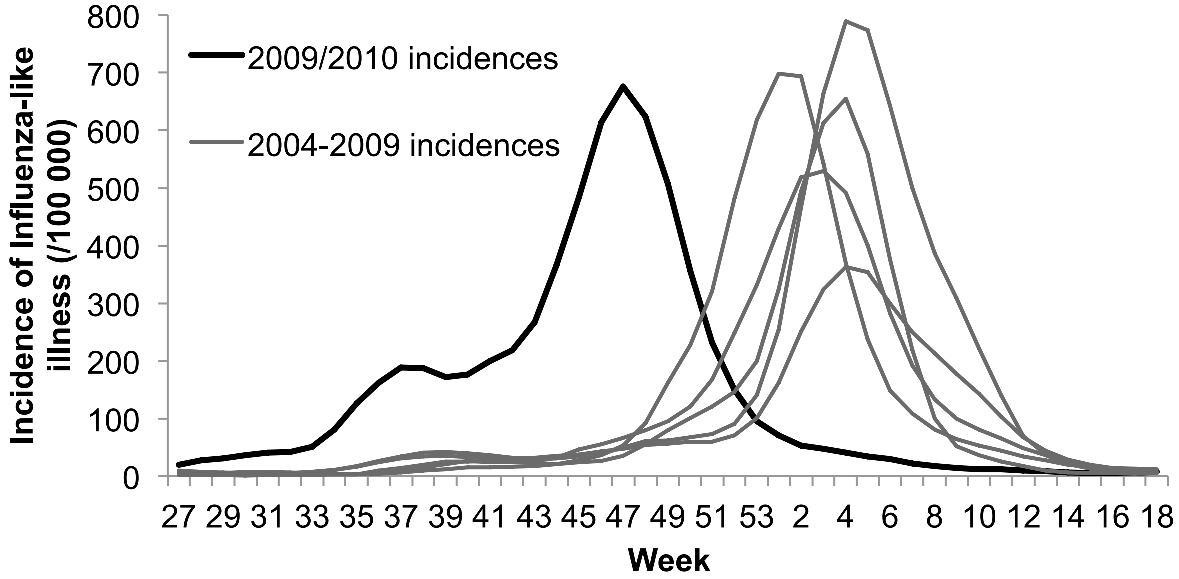

Figure S1: Incidences of influenza-like illness in 2009-2010 and in the previous year (2004-2009) for France. Observed incidences of influenza in 2009-2010 (black lines) arrived sooner than previous years. The plot shows smoothed data.
