# Supplementary material for: Potential Impact of Influenza A/H1N1 Pandemic and Hand-Gels on Acute Diarrhea Epidemic in France
Source: PLoS One. 2013 Oct 4;8(10):e75226. doi: 10.1371/journal.pone.0075226 (PMC3790785; doi:10.1371/journal.pone.0075226)
Supplement: Figure S2 — Incidences of acute diarrhea in 2009–2010 and in the previous year (2004–2009) in France. Observed incidence of acute diarrhea in 2009–2010 (black line) peaks later and higher than the incidence from previous years. The plot shows smoothed data. (PDF) [file pone.0075226.s002.pdf]

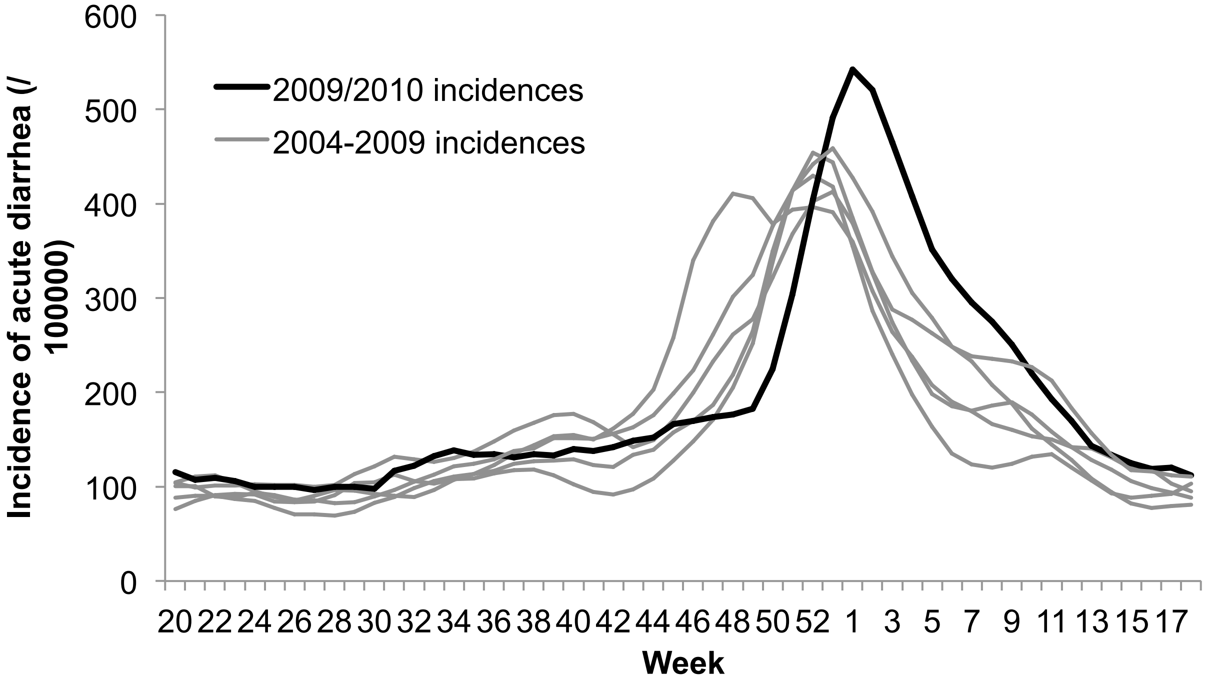


**Figure S2: Incidences of acute diarrhea in 2009-2010 and in the previous year (2004-2009) in France**. Observed incidence of acute diarrhea in 2009-2010 (black line) peaks later and higher than the incidence from previous years. The plot shows smoothed data.
