# Supplementary material for: Potential Impact of Influenza A/H1N1 Pandemic and Hand-Gels on Acute Diarrhea Epidemic in France
Source: PLoS One. 2013 Oct 4;8(10):e75226. doi: 10.1371/journal.pone.0075226 (PMC3790785; doi:10.1371/journal.pone.0075226)
Supplement: Figure S4 — Incidences of acute diarrhea in 2008–2009 for France. Observed incidences are shown with black lines while expected incidences are shown with gray lines along their 95% confidence interval in dashed lines. Expected incidences are based on 2004–2008 data. The comparison shows that estimations of the 2008–2009 incidences are consistent with observations. The plot shows smoothed data. (PDF) [file pone.0075226.s004.pdf]

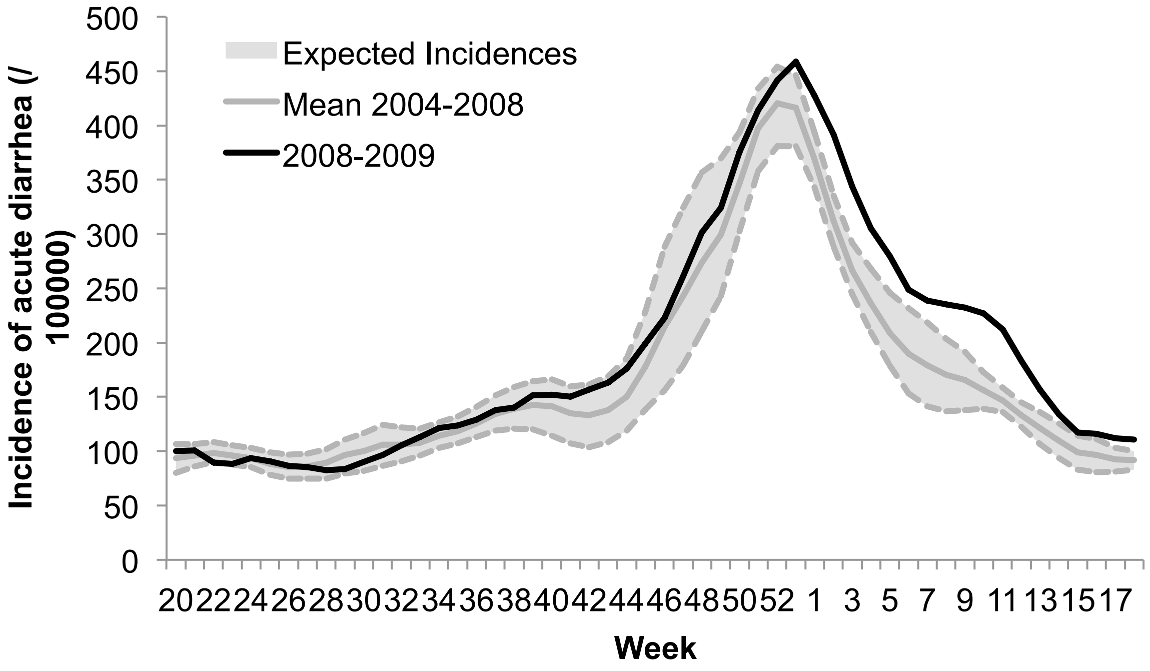

Figure S4: Incidences of acute diarrhea in 2008-2009 for France. Observed incidences are shown with black lines while expected incidences are shown with gray lines along their 95% confidence interval in dashed lines. Expected incidences are based on 2004-2008 data. The comparison shows that estimations of the 2008-2009 incidences are consistent with observations. The plot shows smoothed data.
